# Human BDCA2^+^CD123^+^CD56^+^ dendritic cells (DCs) related to blastic plasmacytoid dendritic cell neoplasm represent a unique myeloid DC subset

**DOI:** 10.1007/s13238-015-0140-x

**Published:** 2015-03-18

**Authors:** Haisheng Yu, Peng Zhang, Xiangyun Yin, Zhao Yin, Quanxing Shi, Ya Cui, Guanyuan Liu, Shouli Wang, Pier Paolo Piccaluga, Taijiao Jiang, Liguo Zhang

**Affiliations:** 1 Key Laboratory of Immunity and Infection, Institute of Biophysics, Chinese Academy of Sciences, Beijing, 100101 China; 2 Key Laboratory of Protein and Peptide Pharmaceuticals, Institute of Biophysics, Chinese Academy of Sciences, Beijing, 100101 China; 3 Graduate School of the Chinese Academy of Sciences, Beijing, 100080 China; 4 Department of Cardiology, 306th Hospital of PLA, Beijing, 100101 China; 5 Department of Gynecology and Obstetrics, Beijing Chaoyang Hospital, Capital Medical University, Beijing, 100020 China; 6 Department of Experimental, Diagnostic, and Specialty Medicine, Hematopathology & Hematology Sections, Molecular Pathology Laboratory, S. Orsola-Malpighi Hospital, Bologna University, Bologna, 40126 Italy

**Keywords:** dendritic cells, CD56^+^ DC, pDC, mDC, BPDCN

## Abstract

Dendritic cells (DCs) comprise two functionally distinct subsets: plasmacytoid DCs (pDCs) and myeloid DCs (mDCs). pDCs are specialized in rapid and massive secretion of type I interferon (IFN-I) in response to nucleic acids through Toll like receptor (TLR)-7 or TLR-9. In this report, we characterized a CD56^+^ DC population that express typical pDC markers including CD123 and BDCA2 but produce much less IFN-I comparing with pDCs. In addition, CD56^+^ DCs cluster together with mDCs but not pDCs by genome-wide transcriptional profiling. Accordingly, CD56^+^ DCs functionally resemble mDCs by producing IL-12 upon TLR4 stimulation and priming naïve T cells without prior activation. These data suggest that the CD56^+^ DCs represent a novel mDC subset mixed with some pDC features. A CD4^+^CD56^+^ hematological malignancy was classified as blastic plasmacytoid dendritic cell neoplasm (BPDCN) due to its expression of characteristic molecules of pDCs. However, we demonstrated that BPDCN is closer to CD56^+^ DCs than pDCs by global gene-expression profiling. Thus, we propose that the CD4^+^CD56^+^ neoplasm may be a tumor counterpart of CD56^+^ mDCs but not pDCs.

**Electronic supplementary material:**

The online version of this article (doi:10.1007/s13238-015-0140-x) contains supplementary material, which is available to authorized users.

## INTRODUCTION

Dendritic cells (DCs) are professional antigen-presenting cells found in virtually all tissues. The main function of DCs is to induce T-cell activation, polarization and expansion against invading pathogens while maintaining tolerance to self antigens (Steinman, 2007). Three DC subsets have been identified in the human blood: plasmacytoid dendritic cell (pDC) and two subsets of myeloid DC (mDC) expressing CD1c (BDCA1) and CD141 (BDCA3) respectively (Dzionek et al., 2000; Ziegler-Heitbrock et al., 2010). pDCs are characterized by their specific expression of CD123, BDCA2, BDCA4 and ILT7 (Dzionek et al., 2000; Cao et al., 2006; Rissoan et al., 2002). In addition, pDCs express high levels of interferon regulatory factor 7 (IRF7), Toll like receptor (TLR)-7 and TLR-9. Accordingly, they produce large amounts of type I interferon (IFN-I) upon stimulation with nucleic-acid ligands of TLR7/TRL9 (Siegal et al., 1999; Cella et al., 1999). Transcription factors E2-2 and Spi B are specifically expressed in pDCs and play important roles in their development and maintenance (Cisse et al., 2008; Schotte et al., 2004).

Blastic plasmacytoid dendritic cell neoplasm (BPDCN) is a rare hematological malignancy characterized by the clonal proliferation of pDC-like cells (Facchetti et al., 2008). Neoplastic cells from BPDCN patients express pDC specific molecules, such as BDCA2, CD123, CD4, TCL1, Bcl11A, CD2AP and Spi B (Petrella and Facchetti, 2010). Additionally, BPDCN cells can produce IFN-I upon TLR7 and TLR9 ligands stimulation although at much lower level comparing with pDCs (Chaperot et al., 2001). Interestingly, BPDCN cells also express CD56, which is not present on the majority of pDCs (Grouard et al., 1997). A small population of DCs in normal or FLT3 mobilized human blood do express CD56, which have been proposed as a pDC subpopulation that gives rise to BPDCN (Petrella et al., 2002; Comeau, 2002).

In this report, we characterized these BDCA2^+^CD123^+^CD56^+^ DCs from human peripheral blood. Although they express some pDC featured molecules, the transcriptomic and functional characterizations indicate that they belong to mDCs rather than pDCs.

## RESULTS

### CD56^+^ DCs are functionally distinct from pDCs

**Figure 1:**
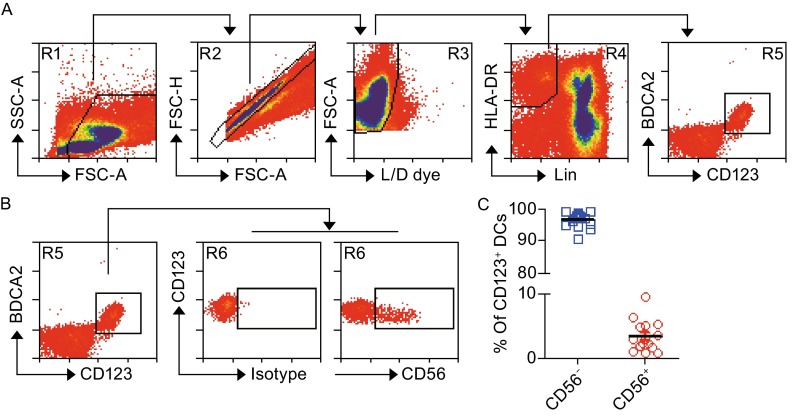
**CD56** ^**+**^ **DCs in human blood**. (A) Human PBMC were first gated based on light scatters, and dead cells were excluded by yellow fluorescent reactive dye (L/D dye), then Lin^−^HLA-DR^+^BDCA2^+^CD123^+^ dendritic cells (DCs) were gated for further analysis. (B) Gated Lin^−^HLA-DR^+^BDCA2^+^CD123^+^ blood DCs were divided into two subsets by their CD56 expression. (C) Summarized data show relative abundance of CD56^−^ and CD56^+^ populations in BDCA2^+^CD123^+^ DCs (Mean ± SD, *n* = 15)

**Figure 2:**
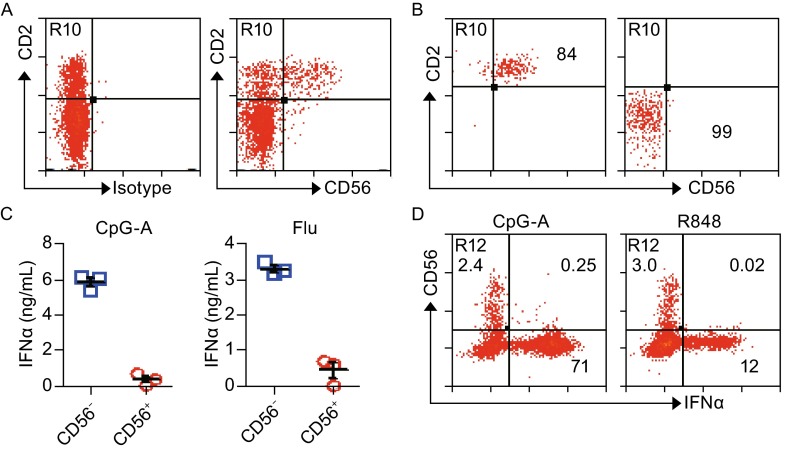
**CD56** ^**+**^ **DCs are functionally distinct from pDCs**. (A) Human PBMC were gated on Lin^−^HLA-DR^+^CD11c^−^CD123^+^ DCs, and the gated DCs were analyzed by their CD2 and CD56 expression. (B) Lineage depleted PBMC were gated on Lin^−^HLA-DR^+^CD11c^−^CD123^+^ DCs, then CD2^+^CD56^+^ and CD2^−^CD56^−^ DCs were sorted by FACS. The purities of sorted cells are shown in each plot. (C) 10,000 purified CD123^+^CD56^−^ and CD123^+^CD56^+^ DCs were stimulated with CpG A or influenza virus (Flu) for 24 h, then interferon α (IFNα) in the supernatants were analyzed by ELISA. Data summarized from 3 independent donors. (D) Lineage depleted PBMCs were stimulated with CpG A or R848 then analyzed by FACS for surface markers and IFNα intracellularly. Lin^−^HLADR^+^CD11c^−^CD123^+^ cells were gated and plotted for CD56 and IFNα. The numbers in each quadrant represent percentage of total gated cells. One out of four independent experiments is shown

BDCA2 and CD123 are commonly used for the identification of human pDCs in the blood and lymphoid tissues. A subpopulation of BDCA2^+^CD123^+^CD56^+^ (CD56^+^) DCs have been reported in human blood and proposed as a pDC subpopulation related to BPDCN (Petrella et al., 2002; Comeau, 2002). We first confirmed the presence of CD56^+^ subset in the HLA-DR^+^, CD11c^−^, BDCA2^+^ and CD123^+^ DCs in human blood (Fig. 1A and 1B). The CD56^+^ subset represents about 5% of the BDCA2 and CD123 double positive cells (Fig. 1B, *n* = 15). It was reported that pDCs can be divided into two populations by their CD2 expression (Matsui et al., 2009). We found that the CD56^+^ DCs universally express CD2 (Fig. 2A).

Next, we purified the CD56^+^ and CD56^−^ cells to compare their IFN-I production by TLR7 or TLR9 stimulation. In order to get higher purity, we added CD2 in our purification protocol, thus CD123^+^CD2^+^CD56^+^ and CD123^+^CD2^−^CD56^−^ DCs were sorted by FACS for further analysis. Usually the purity of CD2^−^CD56^−^ cells was over 95% and the purity of CD2^+^CD56^+^ cells was over 80% with contamination of some CD2^+^CD56^−^ cells (Fig. 2B). High level IFNα production upon TLR7 or TLR9 stimulation is the key characteristic of pDCs. However, purified CD56^+^ DCs produced much less IFNα upon CpG A stimulation comparing to CD56^−^ cells from same donors (around 10%) (Fig. 2C, left panel). Similar results were also observed when the purified DCs were stimulated with influenza virus (Fig. 2C, right panel). In order to exclude the effect of CD56 antibody binding during purification, we also compared the IFNα production capacity of CD56^+^ and CD56^−^ DCs by intracellular staining without prior purification. When enriched DCs were stimulated with CpG A and stained IFNα intracellularly, above 70% of CD56^−^ cells but only 10% CD56^+^ cells expressed IFNα (Fig. 2D, left panel). Similar results were observed by R848, a TLR7 ligand stimulation (Fig. 2D, right panel).

### CD56^+^ DCs clustered together with mDCs but not pDCs by transcriptomic analysis

**Figure 3:**
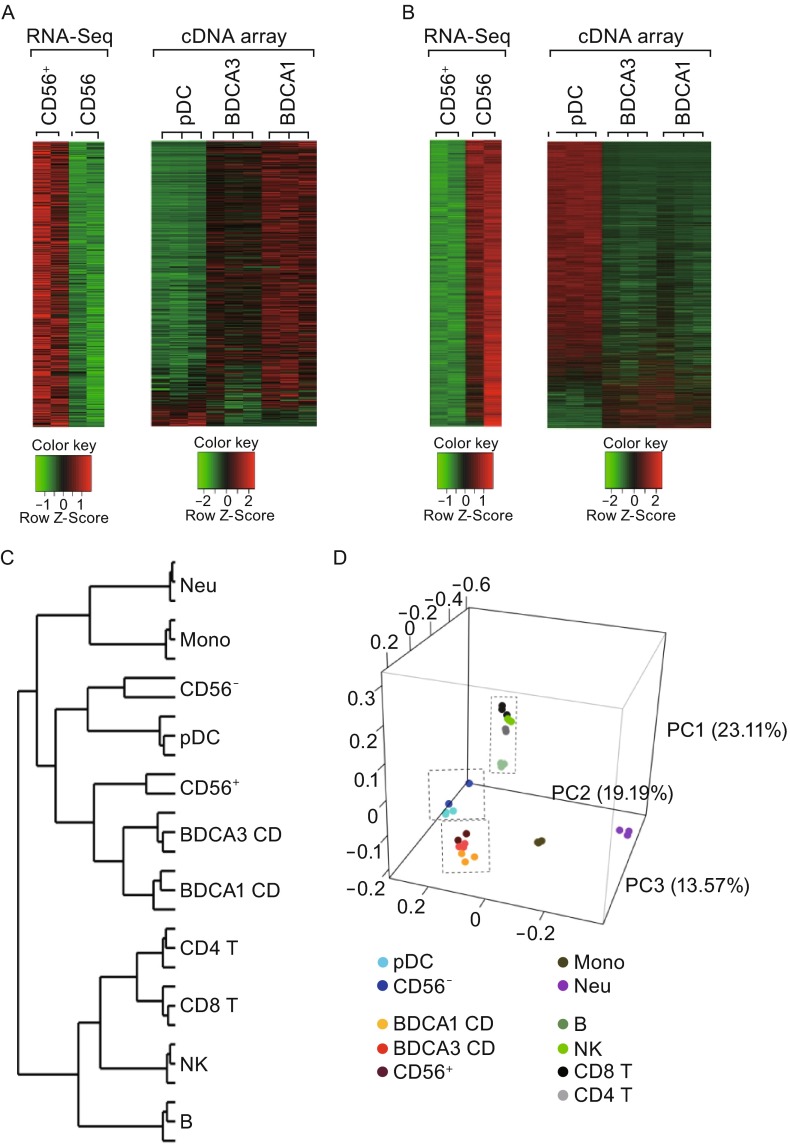
**Comparing gene expression of CD56** ^**+**^ **DCs and other DC subsets**. (A) 648 genes expressed in CD123^+^CD56^+^ DCs at least two fold higher than in CD123^−^ CD56^−^ DCs obtained by RNA-Seq (left panel) and their expressions in pDCs, BDCA3 mDCs and BDCA1 mDCs extracted from cDNA array data (right panel). (B) 1557 lowly expressed genes in CD123^+^CD56^+^ relative to CD123^−^ CD56^−^ DCs (left panel) and their expressions in pDCs, BDCA3 mDCs and BDCA1 mDCs extracted from cDNA array data (right panel). (C) Clustering of CD123^+^CD56^+^ and CD123^−^ CD56^−^ DCs with other immune cell lineages isolated from human peripheral blood based on their gene expression profiles. (D) The principal component analysis (PCA) results. Mono, monocytes; Neu, neutrophils; NK, natural killer cells

In order to compare the gene expression of CD56^+^ DCs and other DC subsets at whole genome level, we analyzed the transcriptome of both CD56^−^ pDCs and CD56^+^ DCs by RNA sequencing (RNA-seq). Next, we compared the transcriptional profile of CD56^+^ DCs with cDNA array data of human DC subsets retrieved from public databases (Robbins et al., 2008). There were 772 genes expressed in CD56^+^ DCs at higher level (over 2 times) than those of CD56^−^ DCs and 2398 genes *vice versa*. Among those, 648 genes highly expressed in CD56^+^ cells (Fig. 3A, left panel) and 1557 lowly expressed ones (Fig. 3B, left panel) were present in the cDNA array data of human blood DC subsets (Robbins et al., 2008). And the names of those differentially expressed genes are provided in Table S1 and S2. Intriguingly, 547 (91%) CD56^+^ DC highly expressed genes present at higher level in mDCs than pDCs based on cDNA array data (Fig. 3A, right panel; Table S1). Similarly, 1292 (83%) CD56^+^ DCs lowly expressed genes presented at lower level in mDCs than pDCs (Fig. 3B, right panel; Table S2).

To clarify the relationship of CD56^+^ DCs and other DC subsets, we normalized our RNA-Seq data with cDNA data, then did hierarchical clustering with complete linkage and principal component analysis (PCA) (11,372 genes). Both analyses showed that CD56^−^ DCs clustered together with pDCs as expected, while the CD56^+^ DCs clustered together with BDCA3 and BDCA1 mDCs (Fig. 3C and 3D).

### CD56^+^ DCs are functionally analogous to mDCs

**Figure 4:**
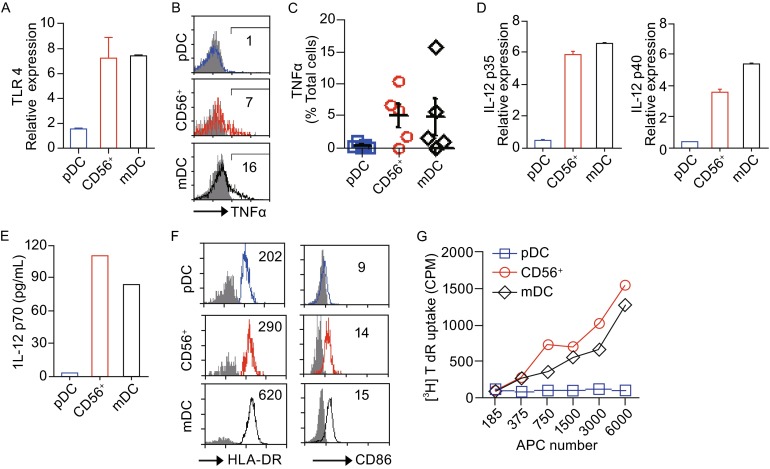
**CD56** ^**+**^ **DCs functionally resemble mDCs**. (A) The expression of TLR4 was quantified by RT-PCR with purified DCs. Error bars represent standard derivation from duplicated PCR samples of the same donor. Data shown are from one representative of 3 independent donors. (B) Lineage depleted PBMC were stimulated with LPS for 6 h, then the stimulated cells were collected and stained with surface markers followed by intracellular staining of TNFα. CD123^+^CD56^−^ (pDC)^,^ CD123^+^CD56^+^ (CD56^+^) and CD123^−^ CD11C^+^ (mDC) in the Lin^−^ HLA-DR^+^ gate were analyzed for TNFα expression. (C) Summarized data of (B) from 5 independent donors. (D) Purified DCs were stimulated with mixture of LPS, CpG and IFNγ for 6 h, the expression of IL-12 p35 and p40 were analyzed by RT-PCR. Data shown are representative of 3 independent experiments. (E) Purified DCs were stimulated mixture of LPS, CpG and IFNγ for 24 h, and the expression of IL-12 p70 were analyzed by ELISA. Data shown are representative of 2 independent experiments. (F) Expression of HLA-DR and CD86 on different blood DC subsets were analyzed by FACS. (G) Purified allogenic naive T cells were cocultured with different numbers of CD123^+^CD56^−^ (pDC, circle)^,^ CD123^+^CD56^+^ (CD56^+^, square) and CD123^−^CD11C^+^ (mDC, triangle) DCs . After 5 days, the cocultured cells were pulsed with 1 mCi [^3^H] thymidine for 18 h before harvest. Data shown are one representative of 4 experiments

TLR expression patterns of pDCs and mDCs are drastically different. pDCs preferentially express TLR7 and TLR9 while mDCs express higher levels of TLR2 and TLR4 (Liu 2005; Cerboni et al., 2013). We found that the expression level of TLR4 (Fig. 4A) and the amount of TNFα produced upon LPS stimulation (Fig. 4B and 4C) were similar between CD56^+^ DCs and mDCs. Moreover, CD56^+^ DCs could produce IL-12 at a comparable level to mDCs at both RNA (Fig. 4D) and protein level (Fig. 4E). While pDCs did not produce IL-12 (Fig. 4D and 4E) as previously reported (Kadowaki et al., 2001).

Different from pDCs, mDCs could prompt T-cell proliferation without prior stimulation or activation. In order to further characterize the relationship between CD56^+^ DCs, pDCs and mDCs, we examined more gene expression that related to antigen presentation. MHC II molecules and CD86 were highly expressed on CD56^+^ DCs compared to pDCs at RNA level (Table S3). These were confirmed at protein level by FACS analysis (Fig. 4F). Gamma-interferon-inducible lysosomal thiolreductase (GILT) is an essential enzyme for antigen processing, (Singh and Cresswell 2010; Maric et al., 2001) and we found that GILT and cathepsins were expressed at much higher levels in CD56^+^ DCs than pDCs (Table S3). We next examined the antigen presentation by CD56^+^ DCs, pDCs and mDCs. Allogeneic naïve T cells were co-cultured with purified DCs and analyzed by tritium incorporation. The CD56^+^ DCs stimulated comparable T-cell proliferation to mDCs, while pDCs did not induce significant T-cell expansion (Fig. 4G).

### CD56^+^ DCs are unique mDCs mixed with some pDC features

**Figure 5:**
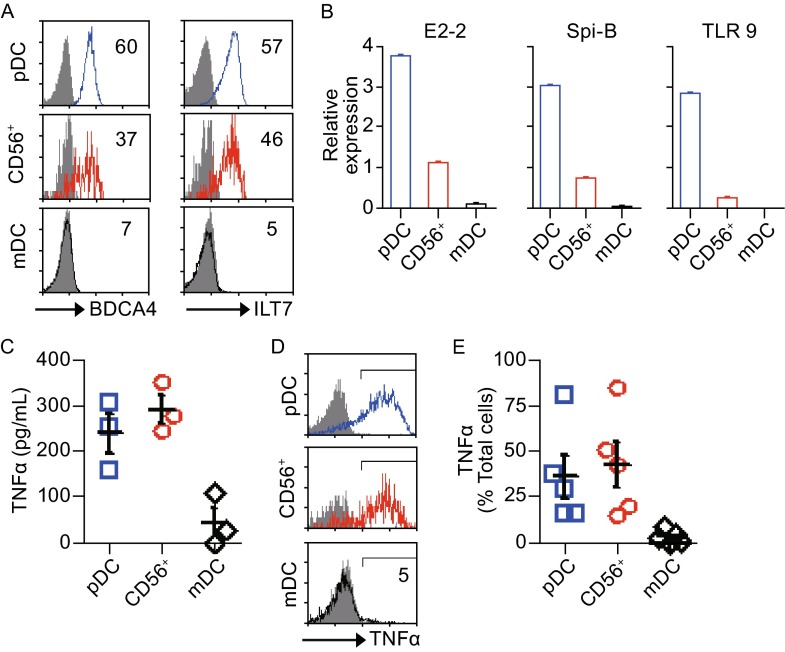
**CD56** ^**+**^ **DCs adopt certain characteristics attributed to pDCs**. (A) The expression of BDCA4 and ILT7 in different DC subpopulations from PBMC were analyzed by FACS. Data shown are one representative of 3 donors. (B) CD123^+^CD56^−^ (pDC), CD123^+^CD56^+^ (CD56^+^) and CD123^−^CD11C^+^ (mDC) were purified from PBMC, E2-2, Spi-B and TLR9 expression in those three DC populations were analyzed by RT-PCR. Error bars represent standard derivation from duplicated PCR samples of same donor. Data shown are one representative of 3 donors. (C) Purified DCs were stimulated with CpG B for 24 h, and the expression of TNFα were analyzed by ELISA. Data shown are summarized from 3 independent experiments. (D) Lineage depleted PBMCs were stimulated with CpG B for 6 h, then stimulated cells were collected and stained with Lin, HLA-DR, CD123, CD11C and CD56 followed by intracellular staining of TNFα. The Lin^−^ HLA-DR^+^ cells were divided into CD123^+^CD56^−^ (pDC), CD123^+^CD56^+^ (CD56^+^) and CD123^−^ CD11C^+^ (mDC) subsets, TNFα expression in each DC subset were analyzed. (E) Summarized data of (D) from 5 independent donors

CD56^+^ DCs expressed BDCA2 and CD123, which were considered as hallmarks of pDCs (Fig. 1B). In order to further characterize the relationship between CD56^+^ DCs and pDCs, we examined more pDC-specific genes at both RNA and protein levels in other DC subsets. And we found that BDCA4 and ILT7, two additional pDC specific surface markers, were expressed on CD56^+^ DCs but not mDCs (Fig. 5A). Both E2-2 and SpiB, the two important transcription factors for pDC lineage development, were expressed in CD56^+^ DCs at slightly lower levels than pDCs in the RNA-seq data (Table S4) and confirmed by RT-PCR (Fig. 5B, left two panels). It is worth to pointing out that the expression levels of E2-2 and SpiB in CD56^+^ DCs were much higher than in mDCs (Fig. 5B, left two panels). In addition, pDC highly expressed genes IRF7, BCL11A and CD2AP (Marafioti et al., 2008) were also expressed in CD56^+^ DCs at a slightly lower level compared to pDCs (Table S4). However, other pDC specific genes, such as Granzyme B, TCL1A (Herling et al., 2003), PACSIN1 (Esashi et al., 2012) and BAD-LAMP (Defays et al., 2011), were expressed at least 10 times higher in pDCs than in CD56^+^ DCs in RNA-seq data (Table S4).

We have already shown that CD56^+^ DCs produced very low level IFNα upon CpG stimulation. And this is correlated with the lower expression level of TLR9 in CD56^+^ DCs than pDCs. However, the expression of TLR9 in CD56^+^ DCs is clearly higher than in mDCs (Fig. 5B, far right panel). Interestingly, CD56^+^ DCs produced comparable levels of TNFα to those of pDCs upon CpG B stimulation (Fig. 5C–E). These results argue that CD56^+^ DCs are unique comparing other mDCs by maintaining certain pDC characteristic features.

**Figure 6:**
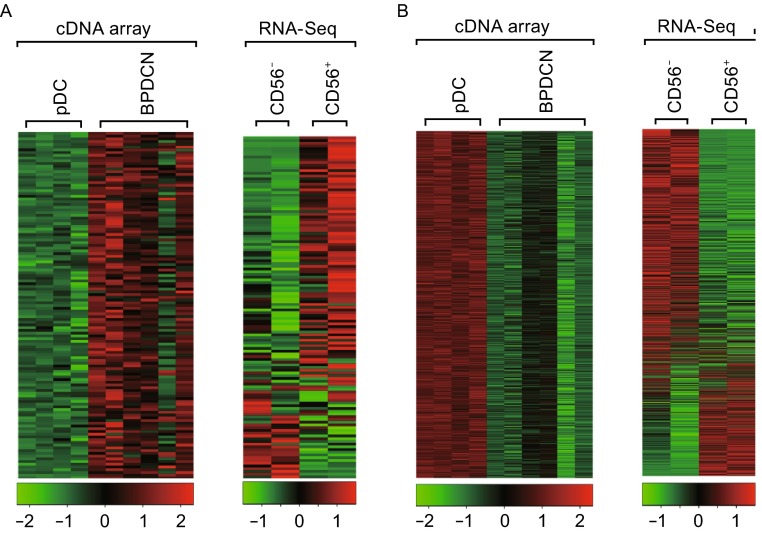
**Global gene expression of BPDCN, pDCs and CD56** ^**+**^ **DCs**. (A) Heat map of 127 genes highly expressed in BPDCN comparing with pDCs by cDNA array (left panel) and their expressions in CD56^+^ DCs relative to CD56^−^ pDCs by RNA-Seq (right panel). (B) Heat map of 1143 genes highly expressed in pDCs comparing with BPDCN by cDNA array (left panel) and their expressions in CD56^+^ DCs relative to CD56^−^ pDCs by RNA-Seq (right panel)

### BPDCN is closely related to CD56^+^ DCs, but not pDCs by global gene expression profiling

In a previous report, we have found that BPDCN shows both myeloid and pDC characteristics (Cerboni et al., 2013). In order to clarify the relationship among BPDCN, pDC and CD56^+^ DCs, we analyzed the differentially expressed genes between BPDCN and pDCs. We identified 127 genes express at higher levels in BPDCN than in pDC in cDNA array data (Fig. 6A, left panel; Table S7), but 93 (73%) of them are highly expressed in CD56^+^ DCs compared to pDCs in our RNA-seq data (Fig. 6A, right panel). Similarly, there are 1143 genes expressed at lower levels in BPDCN cells than in pDCs (Fig. 6B, left panel; Table S8), and 778 (68%) of them are highly expressed in the CD56^+^ DCs than pDCs (Fig. 6B, right panel). The global gene expression profiling data suggests that BPDCN is more closely related to the CD56^+^ DCs than pDCs.

## DISCUSSION

CD4^+^CD56^+^ hematologic malignancy with primary cutaneous presentation has been well documented and is proposed as the malignant counterpart of pDC precursors (Chaperot et al., 2001; Adachi et al., 1994; Brody 1995; Uchiyama 1998; Petrella et al., 1999) and was named as blastic plasmacytoid dendritic cell neoplasm (BPDCN) (Facchetti et al., 2008). Here in this report, we characterized a BDCA2^+^CD123^+^CD56^+^ blood DC subset, which has been considered as a subpopulation of pDCs (Petrella et al., 2002). We demonstrated that the CD56^+^ DCs clustered with mDCs by transcriptional profiling at whole genome level. Accordingly, CD56^+^ DCs responded to TLR4 stimulation and prime naïve T cells without prior stimulation. And this agrees with our previous finding that BPDCN are characterized by their mixed pDC and myeloid signature (Sapienza et al., 2014). However, CD56^+^ DCs are distinguished from other mDCs by expressing pDC specific molecules. Thus, we propose that CD56^+^ DCs represent a unique mDC subset and their function in immune response warrant further characterization.

Besides BPDCN, another pDC related tumoral condition, pDC proliferations in patients with myeloid disorders (PPMD), has also been reported (Petrella and Facchetti 2010). The tumor cells of BPDCN and PPDM share the common characteristic phenotype of pDCs: lin^−^, CD4^+^, CD68^+^, CLA^+^, CD123^+^, BDCA2^+^, Bcl11A^+^ and CD2AP^+^. However, CD56 expression, which is the hallmark of BPDCN, is not detected in PPDM (Petrella and Facchetti 2010; Vermi et al., 2004). Granzyme B (GrB) expresses at high level in pDCs (Rissoan et al., 2002) and is also positive in PPMD (Vermi et al., 2004). However, most reported BPDCN cases are GrB negative (Chaperot et al., 2001; Petrella et al., 2002; Pilichowska et al., 2007; An et al., 2013). Here in this study, we show CD56^+^ DCs express much lower level of GrB compared to pDC (Table S4). We speculate that PPMD may be the tumor counterpart of pDC, while BPDCN may represent the counterpart of CD123^+^CD56^+^ DCs. This hypothesis needs to be further investigated in the future by transcription profiling at whole genome level for both DC subsets and their tumor counterparts.

It is worth pointing out that the human CD123^+^CD56^+^ DCs are similar to a CX_3_CR1^+^CD8α^+^ mouse DC subset, which also exhibits mixed characteristics of pDC and mDC (Ghosh et al., 2010). The CX_3_CR1^+^CD8α^+^ mouse DCs are developmentally dependent on E2-2 and express pDC specific genes (Spi-B, Siglec H, Bst2). However, CX_3_CR1^+^ CD8α^+^ DCs do not produce IFNα upon TLR7 and TLR9 stimulation (Ghosh et al., 2010). The CX_3_CR1^+^CD8α^+^ DC is transcriptionally similar to mouse CD8^−^ mDCs, but not to CD8^+^ mDCs (Ghosh et al., 2010). The developmental relationship between human CD56^+^ DCs and mouse CX_3_CR1^+^CD8α^+^ DCs needs further investigation.

Taken together, we have demonstrated that human CD56^+^ DCs were very close to mDC by both transcriptomic analysis and functional characterization. Thus, we propose that CD56^+^ DC should be classified as mDC, but not pDCs. And we propose that BPDCN should be classified as mDC but not pDC related leukemia.

## MATERIALS AND METHODS

### FACS analysis of DC subsets in human blood

For analysis of DC subsets in human peripheral blood, single cells were first gated by light scatters and dead cells were excluded by yellow fluorescent reactive live/dead cell dye staining (Life Technologies, Carlsbad, USA), then the lineage positive cells were gated out by specific markers (CD3, CD14, CD16, CD19). HLA-DR^+^CD123^+^BDCA2^+^ cells were further divided by their CD2 and CD56 expression. Antibody stained peripheral blood mononuclear cells (PBMC) were analyzed with LSRfortessa (BD Biosciences, Franklin Lakes, NJ, USA) and data were analyzed with Summit 4.3 (DAKO, Denmark). Detailed information of antibodies used for flow cytometry analysis was shown in Table S5.

### DC enrichment and *in vitro* stimulation

DCs were enriched by lineage (CD3, CD14, CD16, CD19) depletion, then lineage negative cells (2 × 10^5^ in 200 μL culture medium) were stimulated with TLR ligands or viruses for 4 h followed by another 2 h in the presence of Golgi Blocker (BD Biosciences, Franklin Lakes, NJ, USA). Cells were stained with surface markers and permeablized and stained with antibodies against various cytokines. TLR ligands were purchased from Invivogen (San Diego, CA, USA) and used at following concentrations: LPS (1 μg/mL), CpG 2216 (2 μg/mL), R848 (2 μg/mL). Heat inactivated influenza virus A/PR8/34 was used at 10 MOI for stimulation.

### DC purification and stimulation

The lineage (CD3, CD14, CD16, CD19, CD20) depleted PBMC were stained with HLA-DR (APC-Cy7), CD2 (PE-Cy7), CD11c (APC), CD56 (PerCP-cy5.5) and CD123 (BV421). Lin^−^HLA-DR^+^CD123^+^CD11c^−^ CD2^+^CD56^+^ and Lin^−^HLA-DR^+^CD123^+^CD11c^−^ CD2^−^CD56^−^ DCs were sorted with BD FACSAriaIII (BD Biosciences, Franklin Lakes, NJ, USA). In some experiments, Lin^−^HLA-DR^+^CD123^−^CD11c^+^ mDC were sorted as control. Purified DCs (1 × 10^4^ in 200 μL culture medium) were stimulated with different TLR ligands at the concentration mentioned above. For IL-12 production, purified DCs were stimulated with LPS, CpG 2006 (1 μmol/L) and IFNγ (50 ng/mL). IFNα, IL-12 and TNFα levels in the supernatant were quantified by ELISA.

### RNA-seq and data analysis

RNA extraction and sequencing were done by BGI Tech (Shenzhen, Guangdong, China). The gene expression level was measured by the number of uniquely mapped reads per kilobase of exon region per million mappable reads (RPKM). The RNA-seq raw data was aligned to the reference genome (hg19) (Trapnell et al., 2009). The gene expressions of other cell types used in our study were obtained from cDNA array data by Scott H Robbins (Robbins et al., 2008). The target gene regions of microarray probes were retrieved from the annotation file for human U133 Plus 2.0 (http://www.affymetrix.com). To compare gene expressions from both RNA-seq and cDNA array experiments, we only considered the reads that have at least one nucleotide overlap with the gene regions targeted by microarray probes. Heat maps were generated with differentially expressed genes between CD2^+^CD56^+^ and CD2^−^CD56^−^ DCs (larger than 2 times difference). Transcriptome comparison of CD2^+^CD56^+^ DCs and other blood cells lineages were carried out with two different mathematical methods, hierarchical clustering with complete linkage (Eisen et al., 1998) and principal component analysis (PCA) (Alter et al., 2000). Detailed methods of RNA-seq and data analysis were provided in the supplemental file.

BPDCN microarray data of 6 cryopreserved tissue samples (Sapienza et al., 2014) by Affymetrix microarray platform were normalized with the rank invariant method using Lumi (Bioconductor) (Du et al., 2008). Statistics for differential expression were applied by Limma (Bioconductor), (Smyth 2004) and we defined BPDCN significant differential expressed genes on normalized data as adjusted *P*-value less than 0.05. One hundred and twenty seven BPDCN highly expressed genes and 1143 BPDCN lowly expressed genes were matched to our RNA-seq datasets of pDC and CD56^+^ DCs (genes with RPKM less than 10 were deleted). Heat maps were displayed using the R software.

### RT-PCR

RNA was extracted from purified DCs and reverse transcribed with Oligo dT primers. Quantitative real time PCR (RT-PCR) was performed using cDNA with EF1α gene as internal control. Primers for RT-PCR are listed in Table S6.

### T-cell proliferation assay

Human CD4^+^CD45RA^+^ naïve T cells were purified from PBMC by negative selection with magnetic beads (Miltenyi Biotech, Germany). Different numbers of FACS purified DCs were cultured with 5 × 10^4^ allogeneic naïve T cells in 96-well round-bottomed plates in 200 μL culture medium. After 5 days of DC and T cell co-culture, cells were pulsed with 1 mCi [^3^H]-thymidine for 18 h before harvest. Radioactive uptake was measured with a TopCount NXT micro-plate scintillation and luminescence counter (PerkinElmer).

## Electronic supplementary material

Below is the link to the electronic supplementary material.

Supplementary material 1 (XLS 173 kb)

Supplementary material 2 (XLS 380 kb)

Supplementary material 3 (DOC 35 kb)

Supplementary material 4 (DOC 34 kb)

Supplementary material 5 (DOC 32 kb)

Supplementary material 6 (DOC 33 kb)

Supplementary material 7 (XLS 42 kb)

Supplementary material 8 (XLS 286 kb)

Supplementary material 9 (DOC 42kb)

## ABBREVIATIONS

BPDCN: blastic plasmacytoid dendritic cell neoplasm
DCs: dendritic cells
IFN-I: type I interferon
IRF7: interferon regulatory factor 7
mDC: myeloid DC
PBMC: peripheral blood mononuclear cell
PCA: principal component analysis
pDC: plasmacytoid DC
TLR: toll like receptor
