# Supplementary material for: Human BDCA2+CD123+CD56+ dendritic cells (DCs) related to blastic plasmacytoid dendritic cell neoplasm represent a unique myeloid DC subset
Source: Protein Cell. 2015 Mar 18;6(4):297–306. doi: 10.1007/s13238-015-0140-x (PMC4383756; doi:10.1007/s13238-015-0140-x)
Supplement: Supplementary file 3 — Supplementary material 3 (DOC 35 kb) [file 13238_2015_140_MOESM3_ESM.docx]

# Supplemental Table 3. Expression of genes related to antigen presentation in CD56^+^ DCs.

|  |  | **Donor1** |  |  | **Donor2** |  |
| --- | --- | --- | --- | --- | --- | --- |
|  | CD56+ | pDC | Ratio CD56^+^/pDC | CD56+ | pDC | Ratio CD56^+^/pDC |
| HLA-DMA | 925 | 555 | **1.7** | 855 | 421 | **2.0** |
| HLA-DMB | 809 | 444 | **1.8** | 662 | 297 | **2.2** |
| HLA-DOA | 130 | 60 | **2.2** | 67 | 56 | **1.2** |
| HLA-DOB | 32 | 2 | **12.8** | 8 | 3 | **2.3** |
| HLA-DPA1 | 3936 | 1061 | **3.7** | 4230 | 889 | **4.8** |
| HLA-DPB1 | 4309 | 1106 | **3.9** | 3513 | 830 | **4.2** |
| HLA-DPB2 | 12 | 3 | **4.3** | 8 | 10 | **0.8** |
| HLA-DQA1 | 811 | 216 | **3.8** | 727 | 197 | **3.7** |
| HLA-DQA2 | 221 | 76 | **2.9** | 48 | 12 | **4.0** |
| HLA-DQB1 | 1587 | 579 | **2.7** | 1159 | 332 | **3.5** |
| HLA-DQB2 | 13 | 3 | **4.4** | 11 | 4 | **2.4** |
| HLA-DRA | 11869 | 4389 | **2.7** | 10794 | 3766 | **2.9** |
| HLA-DRB1 | 3438 | 1259 | **2.7** | 5090 | 1664 | **3.1** |
| HLA-DRB3 | 319 | 116 | **2.8** | 1024 | 343 | **3.0** |
| HLA-DRB4 | 1262 | 491 | **2.6** | 1 | 10 | **0.1** |
| HLA-DRB5 | 2691 | 1015 | **2.7** | 2240 | 824 | **2.7** |
| HLA-DRB6 | 2 | 1 | **2.6** | 0 | 0 | **1.4** |
| CD86 | 89 | 11 | **7.8** | 70 | 7 | **9.9** |
| GILT (IFI30) | 4250 | 21 | **200.3** | 3432 | 38 | **90.7** |
| CTSG | 12 | 0 | **28.3** | 3 | 5 | **0.7** |
| CTSH | 397 | 23 | **17.6** | 365 | 5 | **74.1** |
| CTSW | 74 | 17 | **4.3** | 99 | 14 | **6.9** |

Notes: RNA-seq data from 2 independent donors. The numbers in the table represent reads per kilobase per million mapped reads (RPKM) . pDC, BDCA2^+^CD56^-^pDCs; CD56^+^, BDCA2^+^CD56^+^  DCs. Ratio (CD56^+^/pDC) represents the RPKM of BDCA2 ^+^ CD56 ^+^  DCs divided by those of BDCA2 ^+^ CD56^-^ pDCs (Bold).
