# Supplementary material for: Human BDCA2+CD123+CD56+ dendritic cells (DCs) related to blastic plasmacytoid dendritic cell neoplasm represent a unique myeloid DC subset
Source: Protein Cell. 2015 Mar 18;6(4):297–306. doi: 10.1007/s13238-015-0140-x (PMC4383756; doi:10.1007/s13238-015-0140-x)
Supplement: Supplementary file 4 — Supplementary material 4 (DOC 34 kb) [file 13238_2015_140_MOESM4_ESM.docx]

# Supplemental Table 4. Expression of pDC-specific genes in CD56^+^ DCs.

|  |  | **Donor1** | | |  | **Donor2** | | |
| --- | --- | --- | --- | --- | --- | --- | --- | --- |
|  |  | CD56^+^ | pDC | Ratio (pDC/CD56^+^) |  | CD56^+^ | pDC | Ratio (pDC/CD56^+^) |
| Transcription factors | E2-2 | 66.1 | 219.9 | **3.3** |  | 81.3 | 200.5 | **2.5** |
|  | SPI-B | 85.4 | 202.2 | **2.4** |  | 111.0 | 111.0 | **1.0** |
| IFN signaling | IRF7 | 202.3 | 789.3 | **3.9** |  | 156.7 | 411.0 | **2.6** |
|  | TLR7 | 1.8 | 58.4 | **32.1** |  | 3.3 | 81.2 | **24.8** |
|  | TLR9 | 8.1 | 98.0 | **12.2** |  | 3.3 | 37.1 | **11.4** |
| Surface markers | IL3RA | 169.8 | 564.7 | **3.3** |  | 178.0 | 319.9 | **1.8** |
|  | BDCA2 | 91.6 | 297.8 | **3.3** |  | 81.0 | 150.8 | **1.9** |
|  | BDCA4 | 5 | 64.1 | **12.7** |  | 5.3 | 48.4 | **9.1** |
|  | ILT7 | 636.5 | 1299.6 | **2.0** |  | 1117.0 | 904.0 | **0.8** |
| Intracellular markers | BCL11A | 5.2 | 21.3 | **4.1** |  | 5.3 | 22.7 | **4.2** |
|  | GZMB | 11.5 | 3719.3 | **324.1** |  | 81.6 | 2094.7 | **25.7** |
|  | TCL1A | 0.8 | 380.2 | **499.2** |  | 6.2 | 183.2 | **29.7** |
|  | PACSIN1 | 0.4 | 30.1 | **69.6** |  | 0.4 | 22.5 | **60.2** |
|  | LAMP5 | 2.9 | 126.6 | **44.3** |  | 3.1 | 76.3 | **24.9** |
|  | CD2AP | 13.2 | 49.5 | **3.8** |  | 18.9 | 73.5 | **3.9** |

Notes: RNA-seq data from 2 independent donors. The numbers in the table represent reads per kilobase per million mapped reads (RPKM) . pDC, BDCA2^+^CD56^-^ pDCs; CD56^+^, BDCA2^+^CD56^+^  DCs. Ratio (pDC/CD56^+^) represents the RPKM of BDCA2 ^+^ CD56^-^ pDCs divided by those of BDCA2 ^+^ CD56 ^+^  DCs (Bold).
