# Supplementary material for: Human BDCA2+CD123+CD56+ dendritic cells (DCs) related to blastic plasmacytoid dendritic cell neoplasm represent a unique myeloid DC subset
Source: Protein Cell. 2015 Mar 18;6(4):297–306. doi: 10.1007/s13238-015-0140-x (PMC4383756; doi:10.1007/s13238-015-0140-x)
Supplement: Supplementary file 5 — Supplementary material 5 (DOC 32 kb) [file 13238_2015_140_MOESM5_ESM.docx]

# Supplemental Table 5. Antibodies used in current study.

| Molecule | Clone | Label | Venter |
| --- | --- | --- | --- |
| CD3 | OKT3 | Non | Biolegend |
| CD14 | HCD14 | Non | Biolegend |
| CD16 | HI16a | Non | Sungene |
| CD19 | HIB19a | Non | Sungene |
| CD3 | UCHT1 | FITC | Biolegend |
| CD14 | M5E2 | FITC | Biolegend |
| CD19 | HIB19 | FITC | Biolegend |
| CD20 | 2H7 | FITC | Biolegend |
| HLA-DR | L243 | APC-Cy7 | Biolegend |
| CD11c | B-ly6 | APC | BD Bioscience |
| CD123 | 6H6 | BV421 | Biolegend |
| CD303 | 201A | PE | Biolegend |
| CD2 | TS1/8 | PE-cy7 | Biolegend |
| CD56 | HCD56 | PerCP-cy5.5 | Biolegend |
| CD304 | AD5-17F6 | PE | Miltenyi |
| CD80 | 2D10 | FITC | Biolegend |
| CD86 | IT2.2 | PE | Biolegend |
| TCL-1 | 1-21 | AF647 | Biolegend |
| IFN-α2b | LT27:295 | PE | Miltenyi |
| TNF-α | MAb11 | APC | Biolegend |
| GrB | GB11 | V450 | BD Bioscience |
