# Supplementary material for: Human BDCA2+CD123+CD56+ dendritic cells (DCs) related to blastic plasmacytoid dendritic cell neoplasm represent a unique myeloid DC subset
Source: Protein Cell. 2015 Mar 18;6(4):297–306. doi: 10.1007/s13238-015-0140-x (PMC4383756; doi:10.1007/s13238-015-0140-x)
Supplement: Supplementary file 6 — Supplementary material 6 (DOC 33 kb) [file 13238_2015_140_MOESM6_ESM.docx]

# upplemental Table 6. Primers for RT-PCR.

| Primer |  | Sequence (5’-3’) | product (bp) |
| --- | --- | --- | --- |
| EF1a | Forward | ATATGGTTCCTGGCAAGCCC | 210 |
|  | Reverse | GTGGGGTGGCAGGTATTAGG |  |
| TLR4 | Forward | TTCTCAACCAAGAACCTGGAC | 198 |
|  | Reverse | CAGGGCTAAACTCTGGATGG |  |
| TLR9 | Forward | GAAGGGACCTCGAGTGTGAA | 269 |
|  | Reverse | GTGCTGCCATGGAGAAGTG |  |
| Spi B | Forward | GACTTACCGTTGGACAGCCC | 111 |
|  | Reverse | CAGCTTCTTGCGAGTCCCT |  |
| E2-2 | Forward | GAGTGTCTCCTCTGGCAGC | 494 |
|  | Reverse | CCATGTGATTCGATGCGTC |  |
| IL-12 P35 | Forward | ATGATGGCCCTGTGCCTTAG | 206 |
|  | Reverse | TCCGGTTCTTCAAGGGAGGA |  |
| IL-12 P40 | Forward | GGAGAGTCTGCCCATTGAGG | 110 |
|  | Reverse | TCTTGGGTGGGTCAGGTTTG |  |
