# Supplementary material for: Human BDCA2+CD123+CD56+ dendritic cells (DCs) related to blastic plasmacytoid dendritic cell neoplasm represent a unique myeloid DC subset
Source: Protein Cell. 2015 Mar 18;6(4):297–306. doi: 10.1007/s13238-015-0140-x (PMC4383756; doi:10.1007/s13238-015-0140-x)
Supplement: Supplementary file 9 — Supplementary material 9 (DOC 42kb) [file 13238_2015_140_MOESM9_ESM.docx]

Supplemental Materials for

Human BDCA2^+^CD123^+^CD56^+^ dendritic cells (DCs) represent a unique myeloid DC subset

Haisheng Yu **^1,3 *^**, Peng Zhang**^2^**^,^ **^3 *^**, Xiangyun Yin**^1, 3^** , Zhao Yin^4^, Quanxing Shi^4^, Ya Cui**^2^, Liu Guanyuan**^5^, Shouli Wang^4^, Pier Paolo Piccaluga^6^, Taijiao Jiang**^2^**, and Liguo Zhang**^1^**

**^1^**Key laboratory of Immunity and Infection, **^2^**Key Laboratory of Protein and Peptide Pharmaceuticals, Institute of Biophysics, Chinese Academy of Sciences, Beijing 100101, China; ^3^Graduate School of the Chinese Academy of Sciences, Beijing 100080, China; ^4^Department of Cardiology, 306th hospital of PLA, Beijing 100101, China; ^5^Department of gynecology and obstetrics, Beijing Chaoyang Hospital, Capital Medical University, Beijing, China; ^6^Department of Experimental, Diagnostic, and Specialty Medicine, Hematopathology & Hematology Sections, Molecular Pathology Laboratory, S. Orsola-Malpighi Hospital,

Bologna University, Bologna, Italy.

**^*^**Address correspondence and reprint requests to Liguo Zhang ( [liguozhang@ibp.ac.cn](mailto:liguozhang@ibp.ac.cn)) and Taijiao Jiang ([taijiao@moon.ibp.ac.cn](mailto:taijiao@moon.ibp.ac.cn))

# This PDF file includes:

Supplemental Methods.

Table S5-S8

Table S1-S4 are provided as separated files.

# Supplemental Methods.

## cDNA library preparation and sequencing

RNA was extracted from sorted DC subsets,10 ng total RNA from each sample was used for purification, fragmentation and amplification. Beads containing oligo (dT) were used to isolate poly(A) mRNA from total RNA. First-strand cDNA was synthesized with fragmented RNA and random hexamer-primers. The second-strand DNA was synthesized using buffer, dNTPs, RNase H and DNA polymerase I. Short double-stranded cDNA fragments were purified with a QIAquick PCR extraction kit (Qiagen, Germany) and end repaired with the addition of an ‘A’ base. Next, the short fragments were ligated to Illumina sequencing adaptors. DNA fragments of a selected size were gel-purified and amplified by PCR. The amplified library was sequenced on an IlluminaHiSeq™ 2000 sequencing machine. The details of the experiment are as follows: Expected library size: 200 bp; Read length: 90 nt; and Sequencing strategy: paired-end sequencing.

## RNA-seq gene expression analysis

The images generated by the sequencers were converted into nucleotide sequences by a base-calling pipeline. The raw reads were saved in the fastq format, and we removed the dirty raw reads prior to analyzing the data. Three criteria were used to filter out dirty raw reads: Remove reads with sequence adaptors; remove reads with unknown nucleotides larger than 5%, remove reads with low quality (more than half of the bases' qualities are less than 5). All subsequent analyses were based on clean reads. At least 4M clean reads were used for subsequent analysis.

The reference sequences used were genome and transcriptome sequences downloaded from the UCSC website (version hg19). Clean reads were respectively aligned to the reference genome and transcriptome using SOAP2 [^1^](#_ENREF_1). No more than 5 mismatches were allowed in the alignment for each read. Reads that could be uniquely mapped to a gene were used to calculate the expression level. The gene expression level was measured by the number of uniquely mapped reads per kilobase of exon region per million mappable reads (RPKM). The formula was defined as below:

$$\mathrm{RPKM}=\frac{{10}^{6}C}{NL/{10}^{3}}$$

in which C was the number of reads uniquely mapped to the given gene; N was the number of reads uniquely mapped to all genes; L was the total length of exons from the given gene. For genes with more than one alternative transcript, the longest transcript was selected to calculate the RPKM. The RPKM method eliminates the influence of different gene lengths and sequencing discrepancies on the gene expression calculation. Therefore, the RPKM value can be directly used for comparing the differences in gene expression among samples.

## Public data source for the datasets analyzed in the paper

Human gene chip compendium dataset were based upon supplemental file generated by Scott H Robbins^[2](#_ENREF_2)^, which consisted of 11,507 ProbeSets for human U133 Plus 2.0, representing individual genes with differential expression between ex vivo isolated cell subsets. Additional RNA-seq raw data of Human M1- and M2-like macrophage (respectively human CD4 and CD8 Primary Cells)have been deposited in the Sequence Read Archive (SRA) database under SRA accessions :SRX134852,SRX134853, SRX134854, SRX134855, SRX134856,SRX1348527(respectively SRA accessions: SRX213998,SRX214323,SRX214322,SRX214324).

## Multi-platform data integration

The RNA-seq raw data analyzed in this paper were respectively aligned to the reference genome (hg19) using TopHat [^3^](#_ENREF_3).We counted the number of uniquely aligned mapped reads overlapping with cDNA array probe regions. Probe region locations were retrieved from probe annotation file for human U133 Plus 2.0 ([http://www.affymetrix.com](http://www.affymetrix.com/)). Based on the read counts we estimated probe region expressions using probe region expression ratio, the formula was defined as below:

$$Probe region expression ratio=\frac{The number of reads overlapping with probe regions}{The number of reads uniquely mapped to all genes}$$

## Heat map of genes upregulated or downregulated in BDCA2^+^CD56^+^ DC relative to pDC

We isolated differential expressed genes between BDCA2^+^CD56^+^ and BDCA2^+^CD56^−^ DCs in the RNA-seq data as mean RPKM value was two fold or greater, with expression above threshold values (RPKM>5). There 772 higher and 2398 lower expressed genes in BDCA2^+^CD56^+^ were identified by these criteria. Of those genes identified in RNA-seq data, 648 upregulated and 1517 downregulated genes could be mapped in the cDNA dataset compile by Robbins et al. [^2^](#_ENREF_2)

We further analyzed these common genes between RNA-seq and cDNA array with a difference in expression between mDC(include BDCA1+DC and BDCA3+DC) and pDC were assessed by an unpaired two-way analysis of variance Student’s t-test (p-value <0.05). . We use our RNA-seq data of BDCA2^+^CD56^+^ and BDCA2^+^CD56^−^ DCs and pDC and mDC Human gene chip compendium dataset generated by Scott H Robbins[^2^](#_ENREF_2) to Heat map of genes significantly upregulated (respectively downregulated) for BDCA2^+^CD56^+^ DCs. The t-test was controlled for multiple hypothesis with a Benjamini and Hochberg FDR of <0.01. We defined pDC and mDC highly-expressed genes on the cDNA array data as mean value was twofold or greater and adjusted p-value <0.01. Among 1557 genes which were common for both pDC upregulated in RNA-Seq and pDC upregulated in cDNA array, there are 915 genes (59%, p <0.05) with the variation trend. Among 648 genes which were common for both CD56+DC upregulated in RNA-Seq and mDC upregulated in cDNA array, there are 440 genes (68%, p <0.05) with the variation trend. The figures were visualized with implemented a function in the R software.

## Hierarchical clustering with complete linkage and Principal component analysis

In order to compare the expression of genes between RNA-seq (probe region expression ratio) and cDNA array, the log values for each of these genes were first normalized to a mean equal to zero and a variance equal to 1, independently in the RNA-seq and cDNA array datasets.

The two normalized datasets (11372 genes) were then pooled and a hierarchical clustering with complete linkage was performed[^4^](#_ENREF_4). The software Cluster and Treeview were used to classify cell subsets according to the proximity of their gene expression pattern as assessed by hierarchical clustering with complete linkage.

We implemented a function in the R software to perform Principal component analysis (PCA) [^5^](#_ENREF_5). This function computes the eigenvalues and eigenvectors of the dataset (11372 genes, NA omitted) using the correlation matrix. The eigenvalues were then ordered from the highest to lowest, indicating their relative contribution to the structure of the data. The projection of each sample defined by components was represented as a dot plot to generate the PCA figures.

## References

1. Li R, Yu C, Li Y, et al. SOAP2: an improved ultrafast tool for short read alignment. *Bioinformatics*. 2009;25(15):1966-1967.

2. Robbins SH, Walzer T, Dembele D, et al. Novel insights into the relationships between dendritic cell subsets in human and mouse revealed by genome-wide expression profiling. *Genome Biol*. 2008;9(1):R17.

3. Trapnell C, Pachter L, Salzberg SL. TopHat: discovering splice junctions with RNA-Seq. *Bioinformatics*. 2009;25(9):1105-1111.

4. Eisen MB, Spellman PT, Brown PO, Botstein D. Cluster analysis and display of genome-wide expression patterns. *Proc Natl Acad Sci U S A*. 1998;95(25):14863-14868.

5. Alter O, Brown PO, Botstein D. Singular value decomposition for genome-wide expression data processing and modeling. *Proc Natl Acad Sci U S A*. 2000;97(18):10101-10106.
